# Statistical analysis plan for Better Evidence for Selecting Transplant Fluids (BEST-Fluids): a randomised controlled trial of the effect of intravenous fluid therapy with balanced crystalloid versus saline on the incidence of delayed graft function in deceased donor kidney transplantation

**DOI:** 10.1186/s13063-021-05989-w

**Published:** 2022-01-18

**Authors:** Elaine M. Pascoe, Steven J. Chadban, Magid A. Fahim, Carmel M. Hawley, David W. Johnson, Michael G. Collins, for the BEST-fluids Investigators and the Australasian Kidney Trials Network

**Affiliations:** 1 Australasian Kidney Trials Network, The University of Queensland, Brisbane, Australia; 2 Department of Renal Medicine, Royal Prince Alfred Hospital, Sydney, Australia; 3 Charles Perkins Centre, The University of Sydney, Sydney, Australia; 4 Department of Nephrology, Princess Alexandra Hospital, Brisbane, Australia; 5 The Translational Research Institute, Brisbane, Australia; 6 Department of Renal Medicine, Auckland District Health Board, Auckland City Hospital, Level 15, Park Road, Grafton, Auckland, New Zealand; 7 Department of Medicine, Faculty of Medical and Health Sciences, University of Auckland, Auckland, New Zealand

**Keywords:** End-stage kidney disease, Delayed graft function, Kidney transplantation, Intravenous fluids, Plasma-Lyte 148, 0.9% saline, Randomised controlled trial, Statistical analysis plan

## Abstract

**Background:**

Delayed graft function, or the requirement for dialysis due to poor kidney function, is a frequent complication of deceased donor kidney transplantation that is associated with inferior outcomes. Intravenous fluids with a high chloride content, such as isotonic sodium chloride (0.9% saline), are widely used in transplantation but may increase the risk of poor kidney function. The primary objective of the BEST-Fluids trial is to compare the effect of a balanced low-chloride crystalloid, Plasma-Lyte 148 (Plasmalyte), versus 0.9% saline on the incidence of DGF in deceased donor kidney transplant recipients. This article describes the statistical analysis plan for the trial.

**Methods and design:**

BEST-Fluids is an investigator-initiated, pragmatic, registry-based, multi-centre, double-blind, randomised controlled trial. Eight hundred patients (adults and children) in Australia and New Zealand with end-stage kidney disease admitted for a deceased donor kidney transplant were randomised to intravenous fluid therapy with Plasmalyte or 0.9% saline in a 1:1 ratio using minimization. The primary outcome is delayed graft function (dialysis within seven days post-transplant), which will be modelled using a log-binomial generalised linear mixed model with fixed effects for treatment group, minimization variables, and ischaemic time and a random intercept for study centre. Secondary outcomes including early kidney transplant function (a ranked composite of dialysis duration and the rate of graft function recovery), treatment for hyperkalaemia, and graft survival and will be analysed using a similar modelling approach appropriate for the type of outcome.

**Discussion:**

BEST-Fluids will determine whether Plasmalyte reduces the incidence of DGF and has a beneficial effect on early kidney transplant outcomes relative to 0.9% saline and will inform clinical guidelines on intravenous fluids for deceased donor kidney transplantation. The statistical analysis plan describes the analyses to be undertaken and specified before completion of follow-up and locking the trial databases.

**Trial registration:**

Australian New Zealand Clinical Trials Registry ACTRN12617000358347. Prospectively registered on 8 March 2017

ClinicalTrials.gov identifier NCT03829488. Registered on 4 February 2019

**Supplementary Information:**

The online version contains supplementary material available at 10.1186/s13063-021-05989-w.

## Background

Delayed graft function (DGF) in kidney transplantation is the requirement for dialysis due to poor kidney function in the first week post-transplant [1]. It is a frequent complication of kidney transplants from deceased donors and is associated with inferior outcomes for transplant recipients and higher costs [2–5].

Intravenous (IV) fluids given during and after transplant surgery are critical for the maintenance of intravascular volume and adequate perfusion of the kidney transplant graft. The most commonly used IV fluid is isotonic sodium chloride (‘normal’ or 0.9% saline) [6], which is known to contain a high content of chloride relative to human plasma [7]. Infusion of 0.9% saline causes a predictable hyperchloremic metabolic acidosis [8, 9], especially when administered in high volumes such as those frequently used in transplant patients. Hyperchloremia may increase the risk of acute kidney injury [10–12] and therefore DGF, via renal vasoconstriction and kidney tissue oedema [13]. In addition, acidosis caused by 0.9% saline has been associated with a higher risk of developing hyperkalaemia [14] and therefore the potential for cardiovascular instability and arrhythmias. Whether using a low-chloride balanced crystalloid—designed to more closely reflect the electrolyte concentrations found in human plasma—can reduce the risk of DGF and improve other kidney transplant outcomes is unknown [15].

The BEST-Fluids trial is an investigator-initiated, pragmatic, registry-based, multi-centre, double-blind, randomised controlled trial comparing two approaches to intravenous fluid management in deceased donor kidney transplantation. The primary objective is to compare the effect of Plasmalyte with the effect of 0.9% saline on the incidence of DGF in deceased donor kidney transplant recipients. The secondary objectives are to determine whether perioperative IV therapy using Plasmalyte, compared with 0.9% saline, [1] improves the recovery of graft function in the first week after transplantation, [2] reduces the number of dialysis treatments required, and the duration of dialysis dependence after transplantation, [3] reduces the incidence and severity of hyperkalemia, [4] improves graft survival and death-censored graft survival at 12 months, [5] improves graft function up to 12 months post-transplant, [6] improves health-related quality of life, [7] reduces hospital length of stay and health-related costs, and [8] is cost-effective [16].

The trial was designed to recruit 800 participants (both adults and children) with end-stage kidney disease (ESKD) receiving a deceased donor kidney transplant at participating renal transplant units in Australia and New Zealand. The primary outcome is DGF, defined as requirement for dialysis within 7 days of kidney transplant. This article describes the statistical analysis plan (SAP) for the trial which was written in accordance with the guidelines for SAPs by Gamble et al. [17].

## Methods and design

The full SAP, given in Additional file 1, was written and reviewed by the study statistician and clinical investigators from the BEST-Fluids Trial Steering Committee (TSC). Contributors to the SAP were blinded to treatment allocations and treatment-related study results and will remain so until the data are locked and the final data extracted for analysis. Planned analyses will be performed in accordance with the intention-to-treat principle. Minimization based on three donor characteristics (deceased donor type [donation after brain death, donation after circulatory death], machine perfusion (no, yes), and Australian Kidney Donor Risk Index [KDRI] tertile) and study centre was used to allocate treatments. The main analyses of the primary and secondary outcomes will adjust for these characteristics in addition to ischaemic time.

The primary outcome DGF will be analysed using a log-binomial generalised linear mixed model (GLMM) with fixed effects for treatment group, the three minimisation variables based on donor and transplant characteristics (deceased donor type, machine perfusion, KDRI tertile), and ischemic time, and a random intercept for study centre. The effect of treatment will be reported as a risk ratio (RR, Plasmalyte vs 0.9% saline) and 95% confidence limits from the GLMM analysis. Should the GLMM log-binomial model fail to converge, model simplifying strategies will be adopted in the first instance: adding very small centres to larger ones based on geographic location, excluding machine perfusion as a fixed effect due to the very small number of participants receiving kidneys stored by this method. In the event of intractable convergence issues, a generalised estimating equation (GEE) model (log-binomial; secondarily, Poisson) with exchangeable correlation structure and robust standard errors will be used [18]. Details of supporting and sensitivity analyses of the primary outcome can be found in Additional file 1.

A range of secondary outcomes were measured. Binary secondary outcomes will be analysed and reported using the same approach as for the primary outcome. Other secondary outcomes (continuous, time-to-event, ordinal) will be analysed using similar methods appropriate for the type of outcome.

In addition to detailed descriptions of planned analyses of primary, secondary, exploratory, and safety outcomes, the SAP details planned subgroup analyses where subgroups are formed by the minimization variables and ischaemic time, a comprehensive approach to addressing missing data on the primary and secondary outcomes, and lists changes to outcomes and analyses since publication of the final version of the trial protocol.

## Conclusion

The BEST-Fluids trial will determine whether Plasmalyte has a beneficial effect on early kidney transplant outcomes relative to 0.9% saline via a reduction in the incidence of DGF and other important outcomes. These results will provide definitive data that will inform clinical practice and guidelines on the use of intravenous fluids for deceased donor kidney transplantation.

## Trial status

At the time of initial submission of this article, the BEST-Fluids trial was in the final weeks of follow-up. Recruitment ceased in August 2020. Due to data entry and screening processes at the ANZDATA Registry, data lock is anticipated to be in August/September 2022.

## Supplementary Information

**Additional file 1.** BEST-Fluids SAP v1.00_signed.

## Data Availability

Not applicable since this article describes the statistical methods for the BEST-Fluids trial and does not report any results based on trial data.

## Abbreviations

DGF: Delayed graft function
IV: Intravenous
ESKD: End-stage kidney disease
SAP: Statistical analysis plan
TSC: Trial steering committee
KDRI: Kidney donor risk index
GLMM: Generalised linear mixed model
