# Correction to: Statistical analysis plan for better evidence for selecting transplant fluids (BEST-fluids): a randomised controlled trial of the effect of intravenous fluid therapy with balanced crystalloid versus saline on the incidence of delayed graft function in deceased donor kidney transplantation

**DOI:** 10.1186/s13063-022-06050-0

**Published:** 2022-02-07

**Authors:** Elaine M. Pascoe, Steven J. Chadban, Magid A. Fahim, Carmel M. Hawley, David W. Johnson, Michael G. Collins, for the BEST-fluids Investigators, the Australasian Kidney Trials Network

**Affiliations:** 1 Australasian Kidney Trials Network, The University of Queensland, Brisbane, Australia; 2 Department of Renal Medicine, Royal Prince Alfred Hospital, Sydney, Australia; 3 Charles Perkins Centre, The University of Sydney, Sydney, Australia; 4 Department of Nephrology, Princess Alexandra Hospital, Brisbane, Australia; 5 The Translational Research Institute, Brisbane, Australia; 6 Department of Renal Medicine, Auckland District Health Board, Auckland City Hospital, Level 15, Park Road, Grafton, Auckland, New Zealand; 7 Department of Medicine, Faculty of Medical and Health Sciences, University of Auckland, Auckland, New Zealand

**Correction to: Pascoe (2022)**

**http://orcid.org/10.1186/s13063-021-05989-w**

Following the publication of the original article [1], we were notified that an incorrect additional file was published alongside the paper.

The original article has been corrected.
