# Supplementary material for: Statistical analysis plan for Better Evidence for Selecting Transplant Fluids (BEST-Fluids): a randomised controlled trial of the effect of intravenous fluid therapy with balanced crystalloid versus saline on the incidence of delayed graft function in deceased donor kidney transplantation
Source: Trials. 2022 Jan 18;23:52. doi: 10.1186/s13063-021-05989-w (PMC8764824; doi:10.1186/s13063-021-05989-w)
Supplement: Supplementary file 1 — Additional file 1. BEST-Fluids SAP v1.00_signed. [file 13063_2021_5989_MOESM1_ESM.pdf]

The BEST-Fluids trial AKTN 15.02

STATISTICAL ANALYSIS PLAN (Version 1.01)

**Protocol Title:**	***B****etter* ***E****vidence for* ***S****electing* ***T****ransplant* ***Fluids*** (Version 3.1).
**Short title:**	BEST-Fluids
**Protocol Date:**	2 March 2020
**Trial Registration:**	Australian New Zealand Clinical Trials Registry number ACTRN12617000358347

Australasian Kidney Trials Network
Translational Research Institute, 37 Kent Street, Woolloongabba QLD 4102

DOCUMENT HISTORY

Version	Reason(s) for change	Date
1.00	Initial document	24 August 2021
1.01	Amendments for clarity in response to reviewer comments. Amended sections: 6.5.1, 6.5.2, & 7.2.	17 December 2021

APPROVALS

Principal Investigators & Chairs of the Trial Steering Committee

Name	Signature	Date
Dr Michael Collins	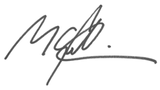	17 December 2021
Prof Steve Chadban	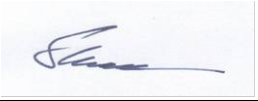	17 December 2021

Trial Statistician, Australasian Kidney Trials Network

Name	Signature	Date
Ms Elaine Pascoe	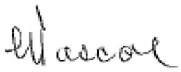	17 December 2021

Chair, Executive Operations Secretariat, Australasian Kidney Trials Network

Name	Signature	Date
Prof Carmel Hawley	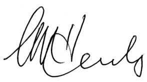	17 December 2021

This *Statistical Analysis Plan is* the property of the University of Queensland through the Australasian Kidney Trials Network. No section of this document may be copied, published, or used for any purpose without appropriate acknowledgement and the prior written permission of the Australasian Kidney Trials Network.

PREFACE

This Statistical Analysis Plan (SAP) describes the planned analyses and reporting for The Australasian Kidney Trials Network (AKTN) protocol 15.02, Better Evidence for Selecting Transplant Fluids (BEST-Fluids): an investigator-initiated, pragmatic, registry-based, multi-centre, double-blind, randomised controlled trial evaluating the effect of Plasmalyte versus 0.9% saline on early kidney transplant function in deceased donor kidney transplantation.

The structure and content of this SAP provide sufficient detail to meet the requirements identified by the International Council for Harmonisation of Technical Requirements for Registration of Pharmaceuticals for Human Use (ICH): Guidance on Statistical Principles in Clinical Trials (1). All work planned and reported for this SAP will follow national and international guidelines for statistical practice (2, 3).

The planned analyses identified in this SAP will be included in future manuscripts. Exploratory analyses not necessarily identified in this SAP may be performed to support planned analyses. Any post-hoc or unplanned analyses not specified in this SAP will be clearly identified as such in the Final Statistical Report (FSR) and manuscripts for publication.

This SAP was written and reviewed by statisticians and clinical investigators from the BEST-Fluids Trial Steering Committee (TSC). All contributors were blinded to treatment allocations and treatment-related study results and will remain so until the central database is locked and the final data extracted for analysis. To ensure and maintain blinding, treatment allocations and statistical code for generating them are stored electronically in a separate location accessible only by a designated un-blinded statistician.

The following documents were reviewed when preparing this SAP:

- Clinical Research Protocol for AKTN Trial Number 15.02 (4).
- Data map, electronic case report forms (eCRFs) and the Data Management Plan for AKTN Trial Number 15.02.
- Operations Manual (Version 4.0) for AKTN Trial Number 15.02.
- Data Safety Monitoring Board (DSMB) Terms of Reference for AKTN Trial Number 15.02.
- ICH Harmonised Tripartite Guideline on Statistical Principles for Clinical Trials (1).
- ICH Harmonised Tripartite Guideline on Estimands and Sensitivity Analysis in Clinical Trials (5).
- ICH Harmonised Tripartite Guideline on Structure and Content of Clinical Study Reports (6).

Readers of this SAP are encouraged to read the Clinical Research Protocol for further details on the conduct of this study and the operational aspects of clinical assessments and timing for completing a patient in this study.

ABBREVIATIONS

ABBREVIATION	DEFINITION
ANZDATA	Australian and New Zealand Dialysis and Transplant Registry
AKTN	Australasian Kidney Trials Network
BMI	Body Mass Index
CKD	Chronic Kidney Disease
CKD-EPI	Chronic Kidney Disease-Epidemiology Collaboration
DBD	Donation after Brain Death
DCD	Donation after Circulatory Death
DGF	Delayed Graft Function
DSMB	Data and Safety Monitoring Board
ECD	Expanded Criteria Donor
eCRF	Electronic Case Report Form
eGFR	Estimated Glomerular Filtration Rate
ESKD	End-Stage Kidney Disease
FSR	Final Statistical Report
GLMM	Generalised linear mixed model
HRC	Health Research Council of New Zealand
ICH	International Council for Harmonisation of Technical Requirements for Registration of Pharmaceuticals for Human Use
ITT	Intention-To-Treat
KRT	Kidney Replacement Therapy
LMM	Linear mixed model
MAP	Mean Arterial Pressure
MAR	Missing at random
MNAR	Missing not at random
MRFF	Medical Research Future Fund of Australia
NHMRC	National Health and Medical Research Council of Australia
SAE	Serious Adverse Event
SAP	Statistical Analysis Plan
TSC	Trial Steering Committee

TABLE OF CONTENTS

DOCUMENT HISTORY *2*

APPROVALS *2*

PREFACE *3*

ABBREVIATIONS *4*

TABLE OF CONTENTS *5*

1. INTRODUCTION *7*

1.1 Background 7

1.2 Study synopsis 7

2. STUDY DESIGN ISSUES *10*

2.1 Overview 10

2.2 Study population 10

2.2.1 Inclusion criteria 10

2.2.2 Exclusion criteria 10

2.3 Study design and treatment allocation 10

2.4. Sample size 11

2.5 Treatment blinding and allocation concealment 12

2.6 Schedule of assessments 12

3. STUDY OUTCOME VARIABLES *13*

3.1 Primary outcome 13

3.2 Secondary outcomes 13

3.3 Exploratory outcomes & sub-studies 15

3.4 Safety outcomes 15

4. SEQUENCE OF PLANNED ANALYSES *16*

4.1 Interim analyses 16

4.2 Blinded review of primary outcome 16

4.3 Final analyses and reporting 17

5. CHANGES TO AND CLARIFICATION OF STATISTICAL INFORMATION IN THE PROTOCOL . *17*

6. ANALYSIS PRINCIPLES *18*

6.1 Intention-to-treat principle and analysis dataset 18

6.2 Covariate adjustment 18

6.3 Subgroup analyses 18

6.4 Multiple comparisons and multiplicity 18

6.5 Missing data 19

6.5.1 Missing outcome data 19

6.5.2 Missing baseline covariate data 19
7. STATISTICAL METHODS *20*
7.1 Analysis of the primary outcome **20**
7.1.1 Main analysis 20
7.1.2 Supporting and sensitivity analyses 20
7.2 Analysis of secondary outcomes **21**
7.3 Analysis of exploratory outcomes **22**
7.4 Analysis of safety outcomes **23**
7.5 Data manipulation and computing **23**
8. REPORTING *23*
8.1 Trial profile **23**
8.2 Protocol deviations **23**
8.3 Patient characteristics and baseline comparisons **23**
8.4 TABLES, LISTINGS, AND FIGURES (TLFs) **24**
8.4.1 Planned tables 24
8.4.2 Planned listings 25
8.4.3 Planned figures 25
8.4.4 Supplementary TLFs 25
8.5 General reporting conventions **25**
8.6 Statistical summary conventions **26**
9. REFERENCES *26*
10. APPENDIX *28*
10.1 Equations for estimating GFR **28**

1. INTRODUCTION

1.1 Background

End-stage kidney disease (ESKD) imposes a large and growing healthcare burden on patients, carers and healthcare systems in Australia, New Zealand (NZ), and the world. ESKD is fatal unless treated with dialysis or transplantation. Kidney transplantation is the best treatment for ESKD and offers improved survival and quality of life at significantly lower cost than dialysis (7, 8).

The key problems facing kidney transplantation are a shortage of donor organs and premature transplant failure. Given the benefits of transplantation, political and medical initiatives including a National Reform Agenda were implemented in Australia between 2009-2015 and produced an increase in the organ donation rate by 53% over that period. Similar initiatives have been implemented in New Zealand. While such reforms have directly addressed, though not solved, the shortage of donor organs, the increased donation rate has been achieved in part by broadening the criteria for organ donation, including older donors and donors with medical co-morbidities. These donor factors have implications for short and long-term graft function. Specifically, the proportions of expanded criteria donors (ECD; donors of older age with co-morbidities) and donors after circulatory (or cardiac) death (DCD) have both increased substantially (9). Kidney transplants from ECD and DCD kidney donors have a higher risk of poor initial kidney transplant function, known as delayed graft function (DGF), and related to this and other factors, subsequent graft dysfunction, graft failure and recipient mortality (10-13). The key causes of premature transplant failure are chronic rejection and premature patient death (14). Beyond the first year after transplantation, transplant failure occurs at a rate of 4-5% per annum; current therapies have failed to impact this. Strategies to reduce transplant failure rates are urgently required.

DGF, the requirement for dialysis or poor kidney transplant graft function early after transplantation, affects 20-50% of deceased donor kidney transplants, and increases the risk of graft failure and patient mortality (15, 16). DGF reflects acute kidney injury caused by ischaemia-reperfusion injury during transplantation, and is driven by donor, recipient and transplant factors. Intravenous fluids are a critical, albeit inexpensive, aspect of care that impacts early transplant function. Currently, 0.9% sodium chloride ('normal' or 0.9% saline) is standard of care (17). However, 0.9% saline may be harmful due to its high chloride content relative to plasma, which causes metabolic acidosis and may promote acute kidney injury, and thus DGF (18, 19). Studies of more physiological, low-chloride, balanced solutions versus normal saline in transplantation have shown reduced acidosis but have been too small to show differences in transplant outcomes (20).

1.2 Study synopsis

The BEST-Fluids trial is an investigator-initiated, pragmatic, registry-based, multi-centre, double-blind, randomised controlled trial comparing two approaches to intravenous fluid management in deceased donor kidney transplantation (21). The trial was designed to recruit 800 participants (both adults and children) with ESKD receiving a deceased donor kidney transplant from participating renal transplant units in Australia and NZ. The primary objective is to determine whether intravenous fluid therapy with a low-chloride, balanced crystalloid solution relative to isotonic sodium chloride reduces the incidence and severity of acute kidney injury and DGF in deceased donor kidney transplant recipients, ultimately

leading to superior long-term outcomes. The primary outcome is DGF, defined as requirement for dialysis within 7 days of kidney transplant.

Figure 1 displays the study schema. Eligible patients (both adults and children) with ESKD receiving a deceased donor kidney transplant were randomly assigned to receive either low-chloride, balanced crystalloid solution, Plasma-Lyte® 148 (Plasmalyte) or Isotonic sodium chloride (0.9% saline).

The planned recruitment target of 800 patients allowed for anticipated rates of treatment drop-out and loss to follow-up (further details are given below). The trial randomised its first participant on 30 January 2018. Due to COVID-19, recruitment was temporarily suspended on 16 March 2020 at all sites and recommenced in May 2020 at NZ sites and June 2020 at Australian sites. Recruitment at all sites ceased in August 2020 after slightly exceeding the recruitment target. The last four participants were randomised on 10 August 2020. In total, 808 participants from 16 kidney transplant centres in Australia (12 centres) and New Zealand (4 centres) were randomised and received a transplanted kidney. An additional 48 patients were randomised and either started surgery but did not actually did not receive a kidney (n=7) or had their transplant surgery cancelled (n=41). The final study visit for the last enrolled participant is expected to be late in August 2021.

Most trial data, including the primary and secondary outcomes and adverse events, are being collected in the Australia and New Zealand Dialysis and Transplant (ANZDATA) Registry. The bulk of these data are being captured in custom-built modules with a limited amount extracted from the standard registry data collection. Additional adverse event data have been extracted from hospital administrative datasets and a final extraction is planned at the end of the trial. The remaining trial data are being captured in a REDCap database, including protocol deviations, serious adverse events and data for an imaging sub-study. From these various sources, the full set of trial data is expected to be clean and available for analysis by August/September 2022. The delay is due to the annual data collection and cleaning processes at the ANZDATA Registry.

Figure 1. Study schema

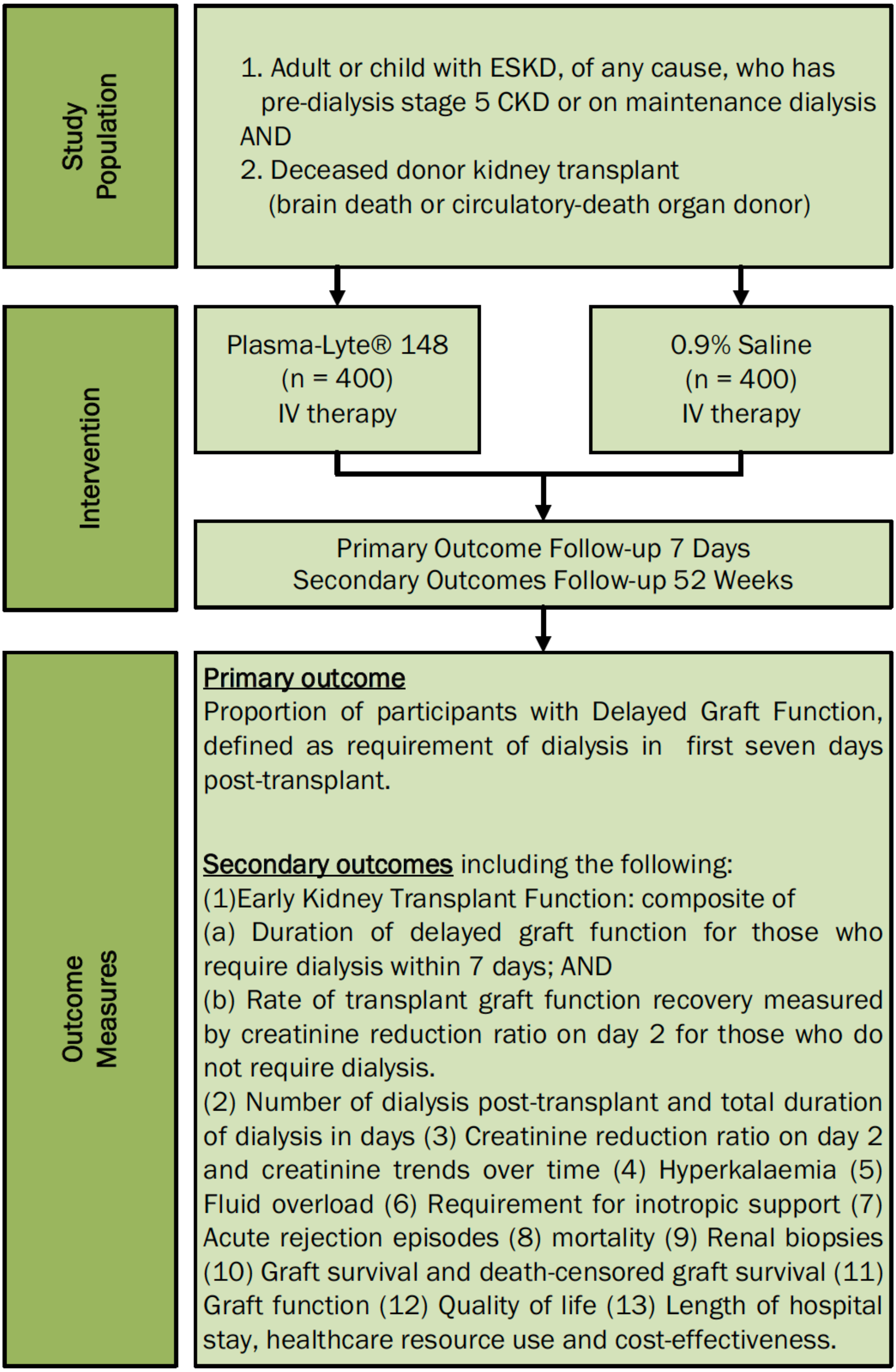

2. STUDY DESIGN ISSUES

2.1 Overview

The study is an investigator-initiated, pragmatic, registry-based, multi-centre, double-blind, randomised controlled trial.

2.2 Study population

The population of interest is adults and children in Australia and NZ with ESKD who are receiving a deceased donor kidney transplant.

2.2.1 Inclusion criteria

Patients were eligible for inclusion in the trial if all of the following criteria were met:

1. Adult or child with ESKD, of any cause, on maintenance dialysis, or who has chronic kidney disease with an estimated glomerular filtration rate of <15 mL/min/1.73m^2 (as determined by the CKD-EPI equation for adults (22) or the bedside Schwartz equation for children (23)).
2. Planned deceased donor kidney transplant from a brain-death (DBD) or circulatory-death (DCD) organ donor expected to occur within the next 24 hours
3. Written informed consent, or consent given by their parent or guardian (if age <18), or other authorised person.

2.2.2 Exclusion criteria

Patients were excluded from the trial if any one of the following conditions was met:

1. Planned live donor kidney transplant (except where this is cancelled in favour of transplantation from a deceased donor)
2. Planned multi-organ transplant (dual or *en-bloc* kidney transplants are not excluded)
3. Be a child with a weight <20 kg, or a child that the treating physician believes should not be included in a study of blinded fluids due to their small body size
4. Known hypersensitivity to the trial fluid preparations or packaging.

2.3 Study design and treatment allocation

Patients were consented then randomised to receive **Plasmalyte** (Plasma-Lyte® 148, approx. pH 7.4, IV infusion, Baxter Healthcare Pty Ltd, Old Toongabbie, NSW; which contains physiologic levels of sodium (140 mmol/L) chloride (98 mmol/L), potassium (5 mmol/L), and magnesium (1.5 mmol/L) buffered with acetate/gluconate), or **0.9% saline** (Sodium chloride 0.9% intravenous infusion BP, Baxter Healthcare; which contains supra-physiologic but isotonic sodium and chloride (both 154 mmol/L)) as a blinded crystalloid fluid solution.

The allocated fluid was administered intravenously for all crystalloid fluid therapy requirements from randomisation onwards throughout the peri-transplant period until 48 hours post-transplant. Treating physicians determined the rate and volume of fluid therapy administered. Non-trial fluid therapies (e.g., blood products) were permitted as per routine

clinical care; treating physicians were asked to avoid using non-trial crystalloids unless there was a compelling, specific clinical indication. All other peri-operative and post-transplant management was as per local standard of care. Dialysis was performed when clinically indicated as per the treating physician.

A covariate-adaptive allocation algorithm was used to assign patients to treatment groups. The algorithm minimised imbalance across treatment groups in the following variables: transplant centre, deceased donor type (DBD, DCD), machine perfusion (no, yes), and Australian Kidney Donor Risk Index (KDRI) tertile. The KDRI is a composite measure of donor quality based on eight donor characteristics known at the time of transplantation (12, 24). Randomisation was implemented using a web-based system called Flexetrials™ which was accessed via the web from the ANZDATA Registry.

2.4. Sample size

The sample size for the BEST-Fluids trial is based on a comparison between two independent groups of the proportions of participants experiencing the primary outcome measure of DGF. The effect size was determined by considering that a relative risk reduction of approximately 25% (relative risk (RR) of 0.75) for the incidence of the primary outcome would be both clinically meaningful and within the range of biological plausibility for the association of DGF with Plasmalyte versus 0.9% saline. The latter is based on trends observed in (1) the Weinberg pilot RCT (25): RR of dialysis within 48h post-transplant of 0.78 (95% confidence interval (CI) 0.47-1.28), and (2) a recently published before-after non-randomised interventional study (26): RR for dialysis within 48h of 0.3 (95% CI 0.10-0.97; adjusted odds ratio 0.14; 95% CI 0.03-0.48), as the most current and relevant data available.

A sample size of 722 participants (361 per group) will have 80% power at a 5% two-sided significance level to show an estimated absolute difference between the groups of 10% (41% versus 31%), being equivalent to a relative risk of DGF for Plasmalyte versus 0.9% saline of 0.76. The combined prevalence of the endpoint of DGF in the first 113 BEST-Fluids trial participants was 36%. Allowing for 4.0 % non-adherence (estimated 2% drop-out from the Plasmalyte group, and 2% drop-in), and up to 1% loss to follow-up for the primary outcome measure (e.g. due to withdrawal of consent within 7 days), an adjusted sample size of 792 participants is required. To allow for fluctuations in these estimates, a total of 800 participants will be recruited.

It is expected that *loss to follow-up* in this study will in reality be close to zero, due to: (1) the very close clinical follow-up that kidney transplant recipients routinely receive; (2) the short timeframe for ascertainment of the primary outcome (7 days post-transplant); and (3) that transplant recipients are a highly motivated group. Indeed, >99% of kidney transplant recipients in Australia/NZ already have complete outcome data recorded in the ANZDATA registry. Furthermore, the study interventions and procedures have been designed in consultation with nephrologists, anaesthetists, intensivists and transplant surgeons from participating centres to ensure acceptability of the protocol, and to minimise *non-adherence* (e.g. drop-in/out due to physicians not using blinded fluids as per protocol).

The assumptions for the sample size calculation (estimates of the combined (blinded) prevalence of the primary endpoint of DGF, rates of loss to follow-up and non-adherence) were reviewed periodically during trial recruitment to confirm that these assumptions remained valid.

2.5 Treatment blinding and allocation concealment

Investigators and participants were blinded to treatment assignment. Biochemistry staff in local laboratories who performed outcome assessments were also blinded to the participant's assigned treatment. To ensure concealment of treatment allocation, randomisation was performed using a central web-based randomisation system called Flexetrials™ administered by the National Health and Medical Research Council (NHMRC) Clinical Trials Centre in Sydney.

2.6 Schedule of assessments

Figure 2. Schedule of study assessments

	STUDY PERIOD								
	Enrolment & Allocation	Transplant and Early Post-operative period				Follow-up			
TIMEPOINT*	*On admission*	*Arrival at recovery*	*Day 1*	*Day 2*	*Day 7*	*Day 28*	*Week 12*	*Week 26*	*Week 52*
ENROLMENT:									
Eligibility screen	X								
Informed consent	X								
Donor details	X								
Baseline characteristics	X								
Allocation	X								
INTERVENTIONS:									
Plasmalyte**	◆———	———	———	———◆					
0.9% Saline**	◆———	———	———	———◆					
ASSESSMENTS:									
Trial fluid volume		X	X	X	X**				
Non-trial fluid type and volume		X	X	X	X**				
Hyperkalemia treatment	X	X	X	X	X**				
Urine output			X	X	X**				
Immunosuppressant drugs	X				X				
Dialysis requirement					X	X	X		
Serum creatinine		X	X	X	X	X	X	X	X
Biopsy requirement					X	X			
Acute rejection					X	X	X	X	X
Mortality and graft survival		X	X	X	X	X	X	X	X
EQ-5D-5L/ EQ-5D-Y	X				X	X	X	X	X
Laboratory assessment		X	X	X					

*Day 1 to week 52 are calculated from the arrival at recovery **Only recorded until 48 hours from arrival at recovery

Windows for study visits are as follows: Day 1 (5:00-9:00 am after arrival in recovery), Day 2 (± 2 hours), Day 7 (± 1 day), Day 28 and Week 12 (± 7 days), Weeks 26 and 52 (± 14 days).

3. STUDY OUTCOME VARIABLES

3.1 Primary outcome

The primary outcome is DGF, defined as receiving treatment with any form of dialysis in the first seven days after transplant. This is a binary outcome that will be categorised as 0=no DGF and 1=DGF. Deaths and graft failures within 7 days post-transplant, of which there will be very few, will be categorised as DGF=1.

3.2 Secondary outcomes

Data are being collected on the following secondary outcomes:

1. *Early Kidney Transplant Function*. This outcome is a ranked composite of two continuous measures of kidney transplant graft function (see Figure 3):
    - Duration of Delayed Graft Function: For participants who require dialysis within seven days post-transplant, the time from transplant to the final dialysis treatment in days - up to 84 days (12 weeks) - will be ranked from best to worst (longer times are worse).
    - Rate of recovery of kidney transplant graft function: For participants who do not require dialysis, graft function assessed using the creatinine reduction ratio on post-transplant day two (CRR2) (27) will be ranked from best to worst (smaller reductions are worse).
        - CRR2 (%) = $([\text{creatinine}_{\text{day 1}}-\text{creatinine}_{\text{day 2}}]*100)/\text{creatinine}_{\text{day1}}$

    The two sets of ordered data will be combined into a single set of ranks measuring best to worst graft function, where the largest increase in CRR2 is ranked highest (best outcome), and the longest time to final dialysis is ranked lowest (worst outcome). Participants with DGF who do not recover graft function by 84 days (12 weeks) will be assigned the worst outcome. Participants with DGF who die without recovering graft function will also be assigned the worst outcome.

Figure 3. Outline of the components of the ranked composite outcome early kidney transplant function (secondary outcome 1)

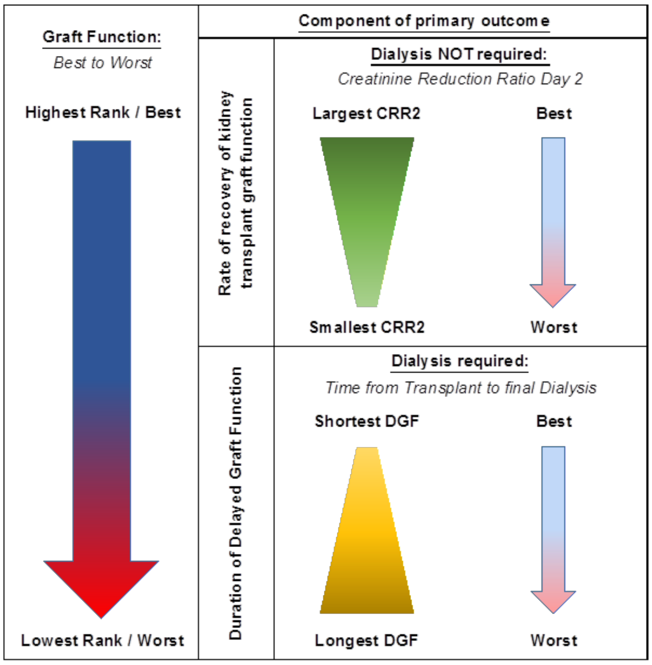

2. Dialysis sessions (for the subset of participants who required dialysis within seven days of transplant):
   a. Number of dialysis sessions in the first 28 days after transplant surgery
   b. Total duration of dialysis, defined as days from transplant surgery to the final dialysis treatment up to 84 days (12 weeks)
3. Serum creatinine reduction (for the subset of participants who did not require dialysis within seven days of transplant):
   a. CRR2
   b. Any decrease in serum creatinine of ≥10% over the first three days post-transplant (where Day 1 serum creatinine is the reference value)
4. Serum creatinine (µmol/L) trends over 52 weeks, measured on 8 occasions (post-op, days 1, 2, 7, & 28, and weeks 12, 26 & 52)
5. Serum potassium (mmol/L), measured on three occasions in the first 48 hours after transplant surgery:
   a. Any serum potassium measurement ≥5.5 mmol/L
   b. Peak potassium level (highest value of three measurements)
6. Treatment for hyperkalaemia (yes or no) where treatment=yes with use of any one or more of dialysis, intravenous calcium, insulin, β-agonists, sodium bicarbonate or ion exchange resins in the first 48 hours after transplant; each of the six component treatments will be separate binary exploratory outcomes
7. Significant fluid overload (yes or no), where overload=yes if weight gain from baseline to day 2 is >5%;
8. Aggregate urine output until day 2 after transplant surgery
9. Any requirement for inotropic support intra-operatively and/or post-operatively;
10. Number of acute rejection episodes in the first 52 weeks after transplant surgery

11. Number of renal transplant biopsies performed within the first 28 days after transplant surgery
12. Mortality (up to 52 weeks) – time to death with censoring at 52 weeks;
13. Graft survival at 52 weeks
    a. Graft survival at 52 weeks
    b. Death-censored graft survival at 52 weeks
    c. Graft survival at 52 weeks with death as a competing risk
14. Graft function (estimated glomerular filtration rate; eGFR) derived from serum creatinine measurements using the CKD-EPI equation (adults) or Bedside Schwartz equation (children) (Appendix 10.1), values for 8 occasions as per serum creatinine (post-op, days 1, 2, 7, & 28, and weeks 12, 26 & 52);
15. Health-related quality of life measured by the EuroQol five dimensions questionnaire (EQ-5D-5L) for adults and the EuroQol five dimensions Youth questionnaire (EQ-5D-Y) for children aged <18 years, measured on 6 occasions (baseline, days 7 & 28, and weeks 12, 26 & 52)
16. Length of stay for the index or transplant hospitalisation, healthcare resource use and cost-effectiveness over 12 months.

3.3 Exploratory outcomes & sub-studies

The trial protocol lists several exploratory outcome variables. Most are in fact entire sub-studies (indicated below). Statistical methods for analysing data from sub-studies will not be described in this SAP.

1. Long term (>12 months) patient and graft outcomes, including mortality, graft survival, and graft function, collected by the ANZDATA registry;
2. [Sub-study] Kinetic estimated GFR (KeGFR) and Creatinine excretion to production ratio (E/eG creatinine) at post-op, 4, 8 and 12 hours, and predictive performance for DGF and graft function;
3. [Sub-study] Change in blood and urine biomarkers of kidney injury, inflammation and cell cycle arrest (NGAL, IL-18, KIM-1, Clusterin, VEGF-A, IGFBP7, TIMP-2) post-transplantation (measured post-op, 4, 8 and 12 hours, and on day 1, 2 and 7), and their relationships with and predictive performance for DGF and graft function;
4. [Sub-study] Intra-renal resistive index derived from Ultrasound Doppler imaging on Day 1-3 post-transplant, including its relationship with graft function and outcomes;
5. [Sub-study] Nuclear medicine scintigraphy measures of graft perfusion measured on Day 1-3 post-transplant, including their relationships with graft function and outcomes.

3.4 Safety outcomes

Safety outcome variables reported by study sites were limited to events considered to be possibly or probably related to study treatment (i.e., between baseline and day 7 after transplant surgery). The following safety outcomes have been recorded:

1. Any serious adverse event (SAE) occurring during the transplant and early post-operative phase (i.e., between baseline and day 7)

   a. Death due to any cause
   b. Any life-threatening event
   c. Any initial or prolonged inpatient hospitalisation
   d. Any persistent or significant disability/incapacity
   e. Any important medical event
   f. Any congenital abnormality/birth defect

2. Relationship of SAE to study treatment
   a. Possible
   b. Probable

Adverse events associated with the index (transplant surgery) hospitalisation and defined by *International Statistical Classification of Diseases and Related Health Problems, Tenth Revision, Australian Modification* (ICD-10) and *Australian Classification of Health Interventions* (ACHI) discharge codes are being obtained from hospital administrative records. SAEs are being categorised as follows:

3. Adverse events of interest:
   a. Ischaemic cardiac event
   b. Arrhythmia
   c. Atrial arrhythmia
   d. Ventricular arrhythmia
   e. Other cardiovascular event
   f. Electrolyte disturbance
   g. Admission to ICU requiring ventilation

4. SEQUENCE OF PLANNED ANALYSES

4.1 Interim analyses

An independent Data and Safety Monitoring Board (DSMB) comprising experts in clinical trials, biostatistics, and nephrology reviewed un-blinded data on participant characteristics, treatment compliance, trial conduct and participant safety. The DSMB held an orientation meeting (25 July 2017) and four data and safety review meetings (4 June 2018 to 27 February 2020) at which un-blinded trial results were assessed. The DSMB recommended continuation of the trial on each occasion. After the first data review meeting the DSMB recommended the collection and reporting of additional safety data based on ICD-10 and ACHI discharge codes. Trial monitoring by the DSMB did not include interim analyses of the primary outcome. Only DSMB members and the statistician compiling closed-session reports for DSMB meetings had access to un-blinded interim data and results.

4.2 Blinded review of primary outcome

Primary outcome DGF data were subjected to blinded review to assess assumptions on which sample size was estimated. These reviews were ad hoc and increased in frequency as the trial approached the recruitment target. The blinded data reviews indicated an overall DGF percentage of 36% that was compatible with the pre-specified clinically important 10% difference in DGF between the two groups (41% vs 31%, assuming an equal number of participants in the two groups).

4.3 Final analyses and reporting

Planned analyses identified in the protocol and in this SAP will be performed only after the last patient has completed the week 52 follow-up assessment visit, the data in both the ANZDATA Registry and the REDCap™ database have been cleaned and locked. There will be no un-blinding and analyses will not commence until this SAP has been approved by the Lead Principal Investigators and Trial Statistician and reviewed and approved by the TSC. Key statistics and trial results from the final analyses will be presented to the TSC for discussion prior to completion of the FSR and subsequent manuscripts. Any post-hoc exploratory analyses performed to provide support for planned analyses but not identified in this SAP will be reported in appendices to the FSR and clearly identified as unplanned analyses. All analyses and their interpretation will be conducted independently of the trial funders: the MRFF of Australia; the Health Research Council (HRC) of New Zealand; and the manufacturer of Plasmalyte, Baxter Healthcare Pty Ltd.

5. CHANGES TO AND CLARIFICATION OF STATISTICAL INFORMATION IN THE PROTOCOL

The following amendments and clarifications have been made to statistical information given in the final version of the trial protocol (4, 21):

1. To allow an intention-to-treat (ITT) analysis of the primary outcome, participants whose grafts failed immediately after transplant and those who died within seven days of their transplant surgery will be categorised as having experienced delayed graft function within seven days of transplant (DGF = 1).
2. Secondary outcome #2 was clarified to indicate that it was relevant to only the subset of participants who required dialysis within seven days of transplant (i.e., DGF = 1, excluding graft failures and death).
3. Secondary outcome #3 was clarified to indicate that it was relevant to only the subset of participants who did not require dialysis (i.e., DGF = 0). Further, the second part was changed from "a decrease in serum creatinine of ≥ 10% on three consecutive days in the first 7 days post-transplant" to "any decrease in serum creatinine of ≥ 10% over the first three days post-transplant".
4. For secondary outcome #13 - graft survival at 52 weeks - a third analysis was proposed after publication of the trial protocol. Previously there were two planned analyses: graft survival which will include death with a functioning graft as an outcome event; and death-censored graft survival where participants who die with a functioning graft will be censored at the time of death. The additional analysis is graft survival with death as a competing risk (28).
5. Two subgroups defined by different dichotomisations of ischaemic time have been added to the list of planned subgroup analyses for a total of five pre-specified subgroups (section 6.3).

6. ANALYSIS PRINCIPLES

6.1 Intention-to-treat principle and analysis dataset

BEST-Fluids is a pragmatic trial comparing two interventions widely used in clinical practice and tests of the effect of treatment on the primary and most secondary outcomes will be conducted according to the intention-to-treat (ITT) principle. Patients who are randomised but have their transplant surgery cancelled and do not receive study fluids prior to the cancellation are deemed not part of the population of interest and will be removed from the study. Patients who receive study fluids but do not receive a kidney will also not be included in the analysis of primary and secondary outcomes but will be included in the analysis of safety outcomes. For the primary outcome, all randomised patients who receive a kidney will be included in the "full analysis set" and analysed in the group to which they were randomly allocated regardless of whether they received the assigned treatment and irrespective of any protocol deviations or violations. The full analysis set for secondary outcomes will be similarly defined except for secondary outcomes #2 and #3 which are specifically aimed at the analysis of data from subsets of participants based on their primary outcome category (DGF vs no DGF). The analysis set for safety outcomes will include all patients who were randomised and received study fluids (i.e., including those who did not receive a transplant).

6.2 Covariate adjustment

The statistical models for comparing Plasmalyte with 0.9% saline on the primary and secondary outcomes will be adjusted for variables used in the minimisation algorithm (deceased donor type [DBD, DCD], machine perfusion [no, yes], Australian Kidney Donor Risk Index [KDRI] tertile), and total ischaemic time. Transplant centre will be a random effect in the statistical models. Additional modelling with *ad hoc* adjustments may be performed where baseline characteristics are not sufficiently balanced across the treatment groups.

6.3 Subgroup analyses

There are five pre-specified subgroup analyses of the primary outcome and the secondary outcome early kidney transplant function. Three subgroups are defined by the donor and transplant variables in the minimisation algorithm: deceased donor type (DBD, DCD), machine perfusion (no, yes), and Australian KDRI (tertiles 1, 2 & 3). Two further subgroups will be defined by ischaemic time: median split of ischaemic time (hours); dichotomised at 14 hours (<14 hours, ≥14 hours) (29). The analysis for each subgroup will be performed by adding the subgroup and its interaction with treatment group as fixed effects to the main statistical model. Treatment group RRs and 95% confidence limits within each sub-group will be reported along with p-values for the treatment x subgroup interaction.

6.4 Multiple comparisons and multiplicity

Multiple hypothesis tests will be performed to assess the effectiveness of Plasmalyte relative to 0.9% saline due to multiple outcomes and pre-specified subgroup analyses. There will be no adjustments for multiplicity as there is a pre-defined hierarchy of importance of study objectives and outcome variables and the influence of individual results on the overall

interpretation of the trial will reflect their level within this hierarchy. Hence, all statistical tests of significance will be two-sided and at the 5% level.

6.5 Missing data

6.5.1 Missing outcome data

The amount of missing data on the primary and most secondary outcomes is anticipated to be substantially less than the 5% rule of thumb sometimes used to justify a complete-case analysis. This expectation is due to the relatively short follow-up periods and/or inclusion of observation time in the definition or analysis of the outcome. Consistent with an expectation of negligible impact of missing data on results, we plan to perform “best-worst” and “worst-best” case sensitivity analyses where data are missing for outcomes measured on a single occasion (30). For the best-worst scenario, missing values in the Plasmalyte group will be allocated the best result (for the primary outcome, DGF=0) and missing values in the 0.9% saline group will be allocated the worst result (for the primary outcome, DGF=1). For the worst-best scenario, the opposite imputations will be performed. Similarly, appropriate best-worst and worst-best imputations will be performed for continuous outcomes measured on a single occasion. After analysis of the best-worst and worst-best datasets, if substantively similar conclusions are reached then no further action will be taken. If substantively different conclusions are reached, then an appropriate (for the outcome) regression analysis will include additional fixed effects which are baseline variables predictive of the outcome and/or missingness on the outcome. If more than 5% of data on a given outcome are missing, then we will perform additional sensitivity analyses based on multiple imputation. This strategy may be applied to the primary outcome and five binary and three continuous secondary outcomes (see Section 7.2).

For repeatedly measured secondary outcomes (serum creatinine, graft function, health-related quality of life), missing outcome data will be addressed using likelihood-based statistical models that allow inclusion of all randomised participants in analyses who have at least one post-randomisation outcome measure. These models assume outcome data are missing at random (MAR). Sensitivity analyses assuming missing not at random (MNAR) and addressing missingness due to participant death will be performed.

6.5.2 Missing baseline covariate data

There will be no missing values on pre-specified covariates included in the main analyses of the primary and secondary outcomes whose collection was required to perform randomisation (i.e., deceased donor type, use of machine perfusion, KDRI tertile). There may be missing values on total ischaemic time and unbalanced baseline variables used as covariates in secondary covariate adjusted analyses of treatment effect. Mean imputation will be used to replace any missing values on these covariates. While mean imputation can bias statistical estimates in observational studies, this is not the case in a randomised trial where randomisation ensures baseline variables are independent of treatment group (31-33). Mean values will be calculated from the non-missing values for the baseline variable using pooled data from both treatment groups. For binary (coded 0 or 1) variables, the imputed mean will be rounded up to 1 or down to 0, whichever is nearest. For computed variables such as Body Mass Index (BMI), mean imputation will be performed at the level of the component variables of height and weight. The number (percentage) of missing values will be reported

by treatment group for all baseline covariates with missing data. To address the possibility that values for a given baseline covariate are not missing completely at random, the statistical models will include a missing value indicator (0=observed, 1=missing) in addition to the mean imputed baseline covariate (31, 33).

7. STATISTICAL METHODS

The objective of all main statistical analyses is to detect a difference between the treatment groups under the research hypothesis that intravenous fluid therapy with Plasmalyte is superior to 0.9% saline.

7.1 Analysis of the primary outcome

The primary goal of the BEST-Fluids trial is to estimate and test treatment differences in DGF, defined as requirement for dialysis within seven days of transplant surgery. The primary outcome will be categorised as 0=no DGF and 1=DGF. Deaths and graft failures (where the kidney transplant graft has been removed) within 7 days post-transplant, of which there are anticipated to be very few, will be categorised as DGF=1. Any participant who withdraws from the study within seven days and does not require dialysis prior to withdrawal will be deemed unclassifiable and excluded from the analysis.

7.1.1 Main analysis

DGF will be analysed using a log-binomial generalised linear mixed model (GLMM) with fixed effects for treatment group, the three minimisation variables based on donor and transplant characteristics (deceased donor type, machine perfusion, KDRI tertile), and ischaemic time, and a random intercept for study centre. The effect of treatment will be reported as a risk ratio (RR, Plasmalyte vs 0.9% saline) and 95% confidence limits from the GLMM analysis. Should the GLMM log-binomial model fail to converge, model simplifying strategies will be adopted in the first instance: adding very small centres to larger ones based on geographic location; excluding machine perfusion as a fixed effect due to the very small number of participants receiving kidneys stored by this method. In the event of intractable convergence issues, a generalised estimating equation (GEE) model (log-binomial; secondarily, Poisson) with exchangeable correlation structure and robust standard errors will be used (34).

7.1.2 Supporting and sensitivity analyses

If there are any unexpected and important differences between treatment groups on baseline variables, a supporting analysis will include these variables as additional fixed effects in the statistical model. A further supplementary analysis will add to the main statistical model a term for the interaction between ischaemic time and deceased donor type. A complete-case sensitivity analysis of the primary outcome will exclude participants who died or their grafts failed within seven days but were classified as DGF=1 for the main analysis. The overall impact of missing data will be assessed via “best-worst” and “worst-best” scenarios as described in section 6.5.1. Non-adherence to use of study fluids is expected to be due to physicians choosing to not use blinded study fluids or inadvertently selecting the wrong box of study fluids, neither of which would necessarily result in treatment non-adherence. The incidence of both and whether there was a discrepancy between randomly allocated and used

fluid will be reported by treatment group. If non-adherence to use of study fluids is more than 4% or there is differential adherence across treatment groups, an adherence-adjusted analysis will be performed.

7.2 Analysis of secondary outcomes

Binary secondary outcome variables with a single planned observation per participant will be analysed using the same approach as for the primary outcome with the effect of treatment reported as a RR and 95% confidence limits. Where binary outcomes are created from continuous data, additional descriptive statistics by treatment group will be reported for the continuous variable. The binary secondary outcome variables are (numbers refer to numeric list in section 3.2):

i. Any decrease in serum creatinine of ≥10% over the first three days post-transplant (outcome 3b)
ii. Any serum potassium measurement ≥5.5 mmol/L in the first 48 hours after transplant surgery (outcome 5a)
iii. Any of 6 treatments for hyperkalaemia in the first 48 hours after transplant surgery (outcome 6)
iv. Significant fluid overload (outcome 7)
v. Any requirement for inotropic support (outcome 9)

Count secondary outcomes will be analysed using GLMM Poisson regression models with fixed and random effects as per the model for the primary outcome. The effect of treatment will be reported as an incidence rate ratio (Plasmalyte vs 0.9% saline) and 95% confidence limits from the GLMM Poison analysis. If there is evidence of over-dispersion, a negative binomial model will be used instead. The count secondary outcome variables are:

i. Number of dialysis sessions in the first 28 days after transplant surgery (outcome 2a)
ii. Number of acute rejection episodes in the first 52 weeks after transplant surgery (outcome 10)
iii. Number of renal transplant biopsies performed within the first 28 days after transplant surgery (outcome 11)

Most time-to-event secondary outcomes will be analysed using Cox regression models with fixed and random effects as per the model for the primary outcome. The effect of treatment will be reported as a hazard ratio (HR. Plasmalyte vs 0.9% saline) and 95% confidence limits from the Cox regression analysis. For the competing risk analysis, a Fine and Gray regression model will be used with the treatment effect reported as a sub-distribution hazard ratio (sdHR) and 95% confidence limits (35). The proportional hazards assumption will be assessed using Schoenfeld residuals. Where necessary, time-dependent treatment effects will be included in the survival models. The time-to-event secondary outcomes are:

i. Total duration of dialysis in days from transplant surgery to the final dialysis treatment up to 84 days (outcome 2b). Death will be assigned the longest duration.
ii. Mortality – time to death with censoring at 52 weeks (outcome 12)
iii. Graft survival at 52 weeks (outcome 13a)
iv. Death censored graft survival at 52 weeks (outcome 13b)
v. Graft survival at 52 weeks with death as a competing risk (outcome 13c)
vi. Length of hospital stay (outcome 16). Death will be assigned the longest duration.

The sole ordinal secondary outcome is early kidney transplant function (outcome 1) which will be analysed using GLMM proportional odds logistic regression with fixed and random effects as per the model for the primary outcome. The effect of treatment will be reported as an odds ratio (Plasmalyte vs 0.9% saline) and 95% confidence limits from the proportional odds logistic GLMM analysis. The proportional odds assumption will be assessed by graphical methods including plotting log odds ratios for multiple cut-offs of the ordinal outcome.

Continuous secondary outcomes measured on a single occasion will be analysed using linear mixed models (LMM) with fixed and random effects as per the model for the primary outcome. The models will not be adjusted for baseline measurements of the variables due to their lack of relevance to post-transplant kidney function. The effect of treatment will be reported as a regression coefficient (Plasmalyte vs 0.9% saline) and 95% confidence limits from the LMM analysis. Assumptions will be assessed graphically plotting residuals against fitted values and using quantile-quantile plots. The relevant continuous secondary outcome variables are:

i. CRR2 (outcome 3a)
ii. Peak potassium level (highest value of three measurements) (outcome 5b)
iii. Aggregate urine output until day 2 after transplant surgery (outcome 8)

Repeatedly measured continuous secondary outcomes will be analysed using linear mixed models for repeated measures (MMRM). The linear MMRM models will have fixed effects for treatment group, categorical time, the treatment-by-time interaction, and baseline measurements of the outcome where available. An unstructured variance-covariance matrix will be used to model the within-patient correlation structure. Treatment group mean differences and 95% confidence limits will be reported for all measurement occasions and across occasions (marginal treatment effect) for the outcome. This specification of the MMRM model is free from assumptions due to the unstructured modelling of treatment effects over time and unstructured within-patient error correlations. Due to this lack of structure, the model is not parsimonious and may suffer from lack of convergence. If the unstructured pattern fails to converge, more parsimonious within-patient error structures will be tested (heterogeneous Toeplitz, Toeplitz, compound symmetric with robust standard errors). The repeatedly measured continuous secondary outcome variables are:

i. Serum creatinine (μmol/L) trends over 52 weeks (8 measurements) (outcome 4)
ii. Graft function (estimated glomerular filtration rate; eGFR) (8 measurements; all time points to be graphed; only weeks 12, 26 & 52 to be included in the MMRM model due to potential association with long-term outcomes (36)) (outcome 14)
iii. Health-related quality of life (baseline + 5 measurements (outcome 15)

7.3 Analysis of exploratory outcomes

Three exploratory outcome variables that are not part of designated sub-studies are long-term (>12 months) mortality, long term graft survival, and long-term graft function (eGFR). Relevant data will be captured by the standard collection of data in the ANZDATA registry. Long-term mortality and graft survival will be analysed by appropriate survival models and long-term graft function by a LMM.

7.4 Analysis of safety outcomes

The relationship of each category of SAE (present versus absent) to treatment group will be summarised by frequencies and percentages and 95% confidence limits for percentages. The relationship between treatment group and SAE relationship to study treatment (possible, probable) will be summarised by frequencies and percentages and 95% confidence limits for percentages. Specific adverse events of interest obtained from hospital records will be similarly described.

7.5 Data manipulation and computing

All data manipulation, tables, figures, listings and analyses will be documented in SAS® or Stata® programs and performed using SAS version 9.4 or later or Stata version 17 or later.

8. REPORTING

All results described above as well as tables, listings and figures (TLFs) listed below will be presented in the FSR.

8.1 Trial profile

All patients who provide informed consent will be accounted for in the FSR. A CONSORT-style flow diagram will illustrate patient progression through the trial from initial screening for eligibility to completion of the final primary outcome assessment. Number (percentage) of participants randomised to each treatment group will be given for all randomised patients along with reasons for study discontinuation (death, withdrawal of consent, at discretion of treating physician, lost to follow-up, other) by treatment group. Reasons for study discontinuation will be reported at seven days (relevant to the primary outcome) and 52 weeks (final study visit).

8.2 Protocol deviations

Protocol deviations falling within the following major categories will be reported: safety, informed consent, eligibility, protocol implementation, and other. Safety sub-categories are: required tests not done, SAE not reported to ethics committee, other. Informed consent sub-categories are: failure to obtain informed consent, non-current consent form used, consent form missing, missing signatures on consent form, other. Eligibility sub-categories are: ineligible participant randomised, other. Protocol implementation sub-categories are: participant seen outside visit window, participant received wrong kit number, missed visit, study sample not collected, assessment done outside window, assessment not done, study fluid not received, other. Sub-categories for other will depend on text responses. All protocol deviations will be reported by treatment group as Table 4.

8.3 Patient characteristics and baseline comparisons

Demographic and other baseline characteristics, including laboratory investigations, will be summarised by assigned treatment group. Categorical variables will be summarised by frequencies and percentages. Percentages will be calculated according to the number of

patients for whom data are available. Where values are missing, the denominator, which will be less than the number of patients assigned to the treatment group, will be reported either in the body or a footnote in the summary table. Continuous variables will be summarised by mean and standard deviation as well as quartiles. Variables to be included in the baseline table are: age (years), sex (female, male), children (≤16 years, >16 years), ethnicity (11 groups), height (cm), weight (kg), body mass index (kg/m^2), smoking status (current, former, never), comorbidities (ischaemic heart disease, congestive heart failure, peripheral vascular disease, cerebrovascular disease, type I diabetes, type II diabetes [not on insulin], type II diabetes [on insulin], chronic lung disease, previous malignancy [non-melanoma skin cancer], previous malignancy [other], other comorbidity), hyperkalaemia (no, yes), treatment for hyperkalaemia (no, yes), year of transplantation (2018, 2019, 2020), dialysis modality before transplant (haemodialysis [HD], peritoneal dialysis [PD], none), previous solid organ transplant (no, yes), graft number (1, 2, 3+), estimated post-transplant survival, panel reactive antibody (peak, current), number of HLA mismatches (0, 1-2, 3-4, 5-6), deceased donor type (DBD, DCD), machine perfusion (no, yes), KDRI tertile (1, 2, 3), total ischaemic time (hours), warm ischaemic time (mins), anastomosis time (mins), induction immunosuppression (Basiliximab, T-cell depletion, B-cell depletion, Intravenous immunoglobulin, other, none), initial oral immunosuppression (Glucocorticoid, Cyclosporin, Tacrolimus, Mycophenolic acid derivative, mTOR inhibitor, other), transplant country/region (Australia [NSW/ACT, QLD, SA/NT, VIC/TAS, WA], New Zealand), centre providing KRT care prior to transplant (transplanting hospital, other).

8.4 TABLES, LISTINGS, AND FIGURES (TLFs)

Templates for all planned TLFs will be in a separate document titled BEST-Fluids SAP TLF. This document will be completed before unblinding of the trial statistician and principal investigators.

8.4.1 Planned tables

The following are planned summary tables:

Table 1. Enrolment by study centre stratified by country
Table 2. Treatment group allocations by study centre
Table 3. Minimisation variables by treatment group
Table 4. Protocol deviations by treatment group (stratified by deviation category)
Table 5. Baseline demographic and clinical characteristics of participants by treatment group
Table 6. Characteristics of donors by treatment group
Table 7. Primary outcome events and percentages by treatment group and RR (95% confidence limits)
Table 8. Secondary outcome events and percentage by treatment group and effect estimates (RR, OR, HR, sdHR) (single measurement outcomes)
Table 9. Serum creatinine treatment group mean values and differences by study visit
Table 10. eGFR treatment group mean values and differences by study visit
Table 11, Quality of life treatment group mean values and differences by study visit
Table 12. Any SAE categories by treatment group
Table 13. SAE relationship to study fluid by treatment group
Table 14. Adverse events of interest by treatment group

8.4.2 Planned listings

The following are planned data and patient listings:

Listing 1. Reasons for participants withdrawing from the study
Listing 2. Deaths and life threatening events

8.4.3 Planned figures

The following are planned summary figures:

Figure 1. Monthly and cumulative entry of participants into the study
Figure 2. Flowchart of patient progression through the study
Figure 3. Cumulative proportion of participants within deciles of early kidney transplant function by treatment group
Figure 4. Kaplan-Meier curves by treatment group for the composite graft survival outcome
Figure 5. Kaplan-Meier curves by treatment group for death censored graft survival
Figure 6. Cumulative incidence functions by treatment group for competing risk analysis of graft survival
Figure 7. Serum creatinine mean values across time by treatment group
Figure 8. eGFR mean values across time by treatment group
Figure 9. Forest plot of subgroup events by treatment group, relative risks (95% confidence limits) and interaction test p-values for subgroup analyses on the primary outcome
Figure 10. Forest plot of subgroup events by treatment group, odds ratios (95% confidence limits) and interaction test p-values for subgroup analyses on early kidney transplant function

8.4.4 Supplementary TLFs

Results from supporting and sensitivity analyses not allocated a specific table number and results from analyses not pre-specified in this SAP will be presented in supplementary tables. Missing data on the primary outcome will be summarised by treatment groups and presented in supplementary tables and figures as appropriate.

8.5 General reporting conventions

All TLFs will be presented in portrait orientation, unless landscape orientation suggests that the information is easier to view. Legends will be used for all figures with more than one variable or item displayed. Figure lines should be wide enough to see the line after being copied.

All titles will be centred on a page. The first title line will be the number of the table, figure, or data listing. The second (and if required, third) line will be the description of the table, figure, or data listing. The ICH numbering convention will be used for all TLFs (6).

All tables, figures, and data listings will have the name of the relevant SAS program and a date-time stamp on the bottom of each output. All analysis programs developed for a table, figure, or data listing will be self-contained to facilitate transfer of programs to multiple computing environments. A separate analysis program will be written to produce each TLF.

8.6 Statistical summary conventions

For tables, sample sizes for each treatment group will be presented as totals in the column header (N=xxx), where appropriate. Sample sizes shown with summary statistics are the number (n) of patients with non-missing values.

Summaries for categorical variables will include only categories that patients had a response in. Percentages corresponding to null categories (cells) will be suppressed. All summaries for continuous variables will include: N, mean, and SD. Other summaries (e.g. median, quartiles, 5%, 95% intervals, coefficient of variation (CV) or %CV will be used as appropriate. All percentages should be rounded and reported to a single decimal place (xx.x%). If percentages are reported as integers, percentages greater than 0% but <1% will be reported as <1%, whereas percentages greater than 99% but <100% will be reported as >99%. A percentage of 100% will be reported as 100%. No value of 0% should be reported. Any computation of percent that results in 0% is to be reported as a blank. Summaries that include p-values will report the p-value to three decimal places with a leading zero (0.001). Small p-values less than 0.001 will be reported as <0.001.

9. REFERENCES

1. ICH. ICH Harmonised Tripartite Guideline: Statistical Principles for Clinical Trials E9. 1998.
2. ASA. Ethical guidelines for statistical practice. Prepared by the Committee on Professional Ethics.1999.
3. ICH. ICH harmonised tripartite guideline: statistical principles for clinical trials E9. 1998.
4. The BEST Fluids Trial Protocol, version 3.1, 2 March 2020.
5. ICH. ICH Harmonised Guideline on Estimands and Sensitivity Analysis in Clinical Trials. 2017.
6. ICH. ICH Harmonised Tripartite Guideline on Structure and Content of Clinical Study Reports E3. 1995.
7. Tonelli M, Wiebe N, Knoll G, Bello A, Browne S, Jadhav D, et al. Systematic review: kidney transplantation compared with dialysis in clinically relevant outcomes. American Journal of Transplantation. 2011;11(10):2093-109.
8. Wyld M, Morton RL, Hayen A, Howard K, Webster AC. A systematic review and meta-analysis of utility-based quality of life in chronic kidney disease treatments. PLoS Medicine. 2012;9(9):e1001307.
9. ANZDATA Registry. 37th Report. Chapter 9: Kidney Donors. Adelaide Australia.; 2015.
10. Collins MG, Chang SH, Russ GR, McDonald SP. Outcomes of transplantation using kidneys from donors meeting expanded criteria in Australia and New Zealand. Transplantation. 2009;87(8):1201-9.
11. Lim WH, McDonald SP, Russ GR, Chapman JR, Ma MK, Pleass H, et al. Association between delayed graft function and graft loss in donation after cardiac death kidney transplants - a paired kidney registry analysis. Transplantation. 2017;101(6):1139-43.
12. Rao PS, Schaubel DE, Guidinger MK, al. e. A comprehensive risk quantification score for deceased donor kidneys: the kidney donor risk index. Transplantation. 2009;88(2):231-6.

13. Summers DM, Johnson RJ, Hudson A, Collett D, Watson CJ, Bradley JA. Effect of donor age and cold storage time on outcome in recipients of kidneys donated after circulatory death in the UK: a cohort study. Lancet. 2013;381(9868):727-34.
14. ANZDATA Registry. 37the Report, Chapter 8: Transplantation. Adelaide, Australia; 2015.
15. Hall IE, Reese PP, Doshi MD, Weng FL, Schroppel B, Asch WS, et al. Delayed Graft Function Phenotypes and 12-Month Kidney Transplant Outcomes. Transplantation 2017;101(8):1913-23.
16. Mallon DH, Summers DM, Bradley JA, Pettigrew GJ. Defining delayed graft function after renal transplantation: simplest is best. Transplantation 2013;96(10):885-9.
17. O'Malley CM, Frumento RJ, Bennett-Guerrero E. Intravenous fluid therapy in renal transplant recipients: results of a US survey. Transplantation Proceedings. 2002;34(8):3142-5.
18. Lobo DN, Awad S. Should chloride-rich crystalloids remain the mainstay of fluid resuscitation to prevent 'pre-renal' acute kidney injury? Kidney International. 2014;86(6):1096-105.
19. Wilcox CS. Regulation of renal blood flow by plasma chloride. Journal of Clinical Investigation. 1983;71(3):726-35.
20. Wan S, Roberts MA, Mount P. Normal saline versus lower-chloride solutions for kidney transplantation. Cochrane Database of Systematic Reviews 2016;Issue 8. Art. No.: CD010741:Art. No.: CD010741.
21. Collins MG, Fahim MA, Pascoe EM, Dansie KB, Hawley CM, Clayton PA, et al. Study Protocol for Better Evidence for Selecting Transplant Fluids (BEST-Fluids): a pragmatic, registry-based, multi-center, double-blind, randomized controlled trial evaluating the effect of intravenous fluid therapy with Plasma-Lyte 148 versus 0.9% saline on delayed graft function in deceased donor kidney transplantation. Trials. 2020;21(1):428.
22. Levey AS, Stevens LA, Schmid CH, Zhang YL, Castro AF, 3rd, Feldman HI, et al. A new equation to estimate glomerular filtration rate. Ann Intern Med. 2009;150(9):604-12.
23. Schwartz GJ, Munoz A, Schneider MF, Mak RH, Kaskel F, Warady BA, et al. New equations to estimate GFR in children with CKD. Journal of the American Society of Nephrology. 2009;20(3):629-37.
24. Clayton PA, Dansie KB, Sypek MP, White S, Chadban S, Kanellis J, et al. External validation of the US and UK kidney donor risk indices for deceased donor kidney transplant survival in the Australian and New Zealand population. Nephrology Dialysis Transplanation. 2019;34(12):2127-31.
25. Weinberg L, Harris L, Bellomo R, F. I, Story D, Eastwood G, et al. Effects of intraoperative and early postoperative normal saline or Plasma-Lyte 148® on hyperkalaemia in deceased donor renal transplantation: a double-blind randomized trial. British Journal of Anaesthesia. 2017;119(4):606-15.
26. Adwaney A, Randall DW, Blunden MJ, Prowle JR, Kirwan CJ. Perioperative Plasma-Lyte use reduces the incidence of renal replacement therapy and hyperkalaemia following renal transplantation when compared with 0.9% saline: a retrospective cohort study. . Clinical Kidney Journal. 2017;10(6):838-44.
27. Rodrigo E, Ruiz JC, Pinera C, Fernandez-Fresnedo G, Escallada R, Palomar R, et al. Creatinine reduction ratio on post-transplant day two as criterion in defining delayed graft function. American Journal of Transplantation. 2004;4(7):1163-9.

28. Sapir-Pichhadze R, Pintilie M, Tinckam KJ, Laupacis A, Logan AG. Survival Analysis in the Presence of Competing Risks: The Example of Waitlisted Kidney Transplant Candidates. American Journal of Transplantation. 2016;16:1958-66.
29. Wong G, Teixeira-Pinto A, Chapman JR, Craig JC, Pleass H, McDonald S, et al. The Impact of Total Ischemic Time, Donor Age and the Pathway of Donor Death on Graft Outcomes After Deceased Donor Kidney Transplantation. Transplantation. 2017;101:1152-8.
30. Jakobsen JC, Gluud C, Wetterslev J, Winkel P. When and how should multiple imputation be used for handling missing data in randomised clinical trials – a practical guide with flowcharts. BMC Medical Research Methodology. 2017;17:162.
31. Groenwald RHH, White IR, Donders ART, Carpenter JR, Altman DG, Moons KGM. Missing covariate data in clinical research: when and when not to use the missing-indicator method for analysis. Canadian Medical Association Journal. 2012;184(11):1265-9.
32. Schemper M, Smith TL. Efficient evaluation of treatment effects in the presence of missing covariate values. Statistics in Medicine. 1990;9:777-84.
33. White IR, Thompson SG. Adjusting for partially missing baseline measurements in randomized trials. Statistics in Medicine. 2005;24:993-1007.
34. Pedroza C, Truong VTT. Estimating relative risks in multicenter studies with a small number of centers — which methods to use? A simulation study. Trials. 2017;18:512.
35. Fine JP, Gray RJ. A proportional hazards model for the subdistribution of a competing risk. Journal of the American Statistical Association. 1999;94:496-509.
36. Clayton PA, Lim WH, Wong G, Chadban SJ. Relationship between eGFR decline and hard outcomes after kidney transplants. Journal of the American Society of Nephrology. 2016;27:3440-2446.

10. APPENDIX

10.1 Equations for estimating GFR

Sex	Serum Creatinine (µmol/L)	Equation†
**CKD-EPI creatinine equation***		
Female	≤62	$144 \times (Scr \times 0.0113/0.7)^{-0.329} \times (0.993)^{Age}$
Female	>62	$144 \times (Scr \times 0.0113/0.7)^{-1.209} \times (0.993)^{Age}$
Male	≤80	$141 \times (Scr \times 0.0113/0.9)^{-0.411} \times (0.993)^{Age}$
Male	>80	$141 \times (Scr \times 0.0113/0.9)^{-1.209} \times (0.993)^{Age}$
**Bedside Schwartz equation**		
Female or male		36.2 x (height/Scr)

†Scr = serum creatinine (µmol/L)**;** Age = age in years; height = height in centimetres.
*Coefficient for black race ([x 1.159]) not included as it is appropriate for African Americans and not Australian Aboriginal and Torres Strait Islanders or New Zealand Māori or Pacific ethnicities.
